# Mechanisms of Smooth Muscle Cell Differentiation Are Distinctly Altered in Thoracic Aortic Aneurysms Associated with Bicuspid or Tricuspid Aortic Valves

**DOI:** 10.3389/fphys.2017.00536

**Published:** 2017-07-25

**Authors:** Elena Ignatieva, Daria Kostina, Olga Irtyuga, Vladimir Uspensky, Alexey Golovkin, Natalia Gavriliuk, Olga Moiseeva, Anna Kostareva, Anna Malashicheva

**Affiliations:** ^1^Laboratory of Molecular Cardiology, Almazov Federal Medical Research Centre Saint Petersburg, Russia; ^2^Department of Medical Physics, Peter the Great Saint-Petersburg Polytechnic University Saint Petersburg, Russia; ^3^Laboratory of Bioinformatics and Genomics, Institute of Translational Medicine, ITMO University Saint Petersburg, Russia; ^4^Faculty of Biology, Saint-Petersburg State University Saint Petersburg, Russia

**Keywords:** thoracic aortic aneurysms, bicuspid aortic valves, tricuspid aortic valves, smooth muscle cells, differentiation

## Abstract

Cellular and molecular mechanisms of thoracic aortic aneurysm are not clear and therapeutic approaches are mostly absent. Thoracic aortic aneurysm is associated with defective differentiation of smooth muscle cells (SMC) of aortic wall. Bicuspid aortic valve (BAV) comparing to tricuspid aortic valve (TAV) significantly predisposes to a risk of thoracic aortic aneurysms. It has been suggested recently that BAV-associated aortopathies represent a separate pathology comparing to TAV-associated dilations. The only proven candidate gene that has been associated with BAV remains *NOTCH1*. In this study we tested the hypothesis that Notch-dependent and related TGF-β and BMP differentiation pathways are differently altered in aortic SMC of BAV- vs. TAV-associated aortic aneurysms. SMC were isolated from aortic tissues of the patients with BAV- or TAV-associated aortic aneurysms and from healthy donors used as controls. Gene expression was verified by qPCR and Western blotting. For TGF-β induced differentiation SMC were treated with the medium containing TGF-β1. To induce proosteogenic signaling we cultured SMC in the presence of specific osteogenic factors. Notch-dependent differentiation was induced via lentiviral transduction of SMC with activated Notch1 domain. *MYOCD* expression, a master gene of SMC differentiation, was down regulated in SMC of both BAV and TAV patients. Discriminant analysis of gene expression patterns included a set of contractile genes specific for SMC, Notch-related genes and proosteogenic genes and revealed that control cells form a separate cluster from both BAV and TAV group, while BAV- and TAV-derived SMC are partially distinct with some overlapping. In differentiation experiments TGF-β caused similar patterns of target gene expression for BAV- and TAV derived cells while the induction was higher in the diseased cells than in control ones. Osteogenic induction caused significant change in *RUNX2* expression exclusively in BAV group. Notch activation induced significant *ACTA2* expression also exclusively in BAV group. We show that Notch acts synergistically with proosteogenic factors to induce *ACTA2* transcription and osteogenic differentiation. In conclusion we have found differences in responsiveness of SMC to Notch and to proosteogenic induction between BAV- and TAV-associated aortic aneurysms.

## Introduction

Thoracic aortic aneurysm (TAA) is a life threatening condition, which is manifested by progressive enlargement of the thoracic aorta due to destructive changes in the aortic wall. Therapeutic agents that may influence the process are absent to date and the only therapeutic decision is a surgical intervention (Davis et al., 2014). Up to 20% of patients with TAA have a positive family history, confirming the autosomal-dominant nature of this disease. The genetic basis of TAA is heterogeneous and involves most importantly genes coding for components of the vascular smooth muscle contractile apparatus, the extracellular matrix of connective tissue and key molecules of the transforming growth factor-β signaling (Luyckx and Loeys, 2015).

TAAs are characterized by extensive loss of smooth muscle cells (SMC) and changes in their functionality (Della Corte et al., 2008; Forte et al., 2013; Phillippi et al., 2014; Malashicheva et al., 2016). At the same time, the role of SMC during development and progression of aortic aneurysms is not well defined. Vascular SMC are characterized by specific molecular markers and contractile functions. Unlike other terminally differentiated muscle cells, vascular SMC have a unique ability to reversibly modify their contractile phenotype to a dedifferentiated state in response to changes in local environmental cues (Owens, 1995; Owens et al., 2004). The molecular basis of SMC differentiation in response to pro-differentiation signals is the expression of several highly specific contractile proteins, including smooth muscle α-actin (SMA), SM22α, and calponin. Abnormal control of the SMC phenotype leads to progression of vascular pathologies, including aneurysms (Ailawadi et al., 2009).

Myocardin (*MYOCD*) is an essential factor for the SMC differentiated phenotype. This transcription cofactor is both necessary and sufficient for the development and differentiation of most SMC (Wang et al., 2003; Long et al., 2008; Miano, 2015). Myocardin is a cardiac and smooth muscle–specific coactivator of serum response factor (SRF) that is able to transcriptionally activate CArG box–containing cardiac and smooth muscle target genes, such as smooth muscle α-actin, or SMA (*ACTA2*), SM22α (*TAGLN*), and calponin (*CNN1*) (Wang et al., 2003; Qiu et al., 2005; Huang et al., 2008; Long et al., 2008).

TGF-beta has been for a long time considered as a key participant of driving SMC differentiation state in the aorta by activating the genetic program that includes a set of SMC differentiation marker genes (Guo and Chen, 2012).

TAA may occur in the presence of a tricuspid or a bicuspid aortic valve (TAV and BAV), respectively. Several lines of evidence suggest that the mechanism of aneurysm development is distinct between the two patient groups (Folkersen et al., 2011; Balistreri et al., 2013; Kjellqvist et al., 2013; Maleki et al., 2013; Phillippi et al., 2014; Paloschi et al., 2015). The genetic background underlying BAV/TAA is clearly not monogenic and complex. So far, a few genes such as *NOTCH1* (Garg et al., 2005; Mohamed et al., 2006; Mckellar et al., 2007; Mcbride et al., 2008; Andreassi and Della Corte, 2016; Forte et al., 2016; Koenig et al., 2017) and *GATA5* (Padang et al., 2012) have been associated with non-syndromic forms of BAV/TAA.

In the vascular system, Notch receptors (Notch1–4) and ligands (Jag1 and 2 and Dll1, 3, and 4) are expressed. Activation of Notch receptors requires binding to a transmembrane ligand presented by neighbor cells. This binding enables a series of cleavage events in the receptor, resulting in the release of the intracellular region of Notch protein (Notch intracellular domain, NICD). NICD, the transcriptionally active form of Notch, translocates to the nucleus where it regulates a broad range of target genes (Andersson et al., 2011). The outcome of Notch activation is cell type and context dependent with multiple combinations of receptors and ligands that transduce different biological effect (Mašek and Andersson, 2017).

Controversy exists regarding the effect of Notch signaling on vascular SMC phenotype. Notch signaling has been linked to smooth muscle differentiation both *in vitro* and *in vivo* (Doi et al., 2006; Noseda et al., 2006; Boucher et al., 2012). At the same time, some data are consistent with a model wherein Notch signaling represses SMC differentiation and maintenance of the contractile SMC phenotype (Sweeney et al., 2004; Morrow et al., 2005; Proweller et al., 2005).

Although, the role of Notch has been extensively studied in the context of development and cancer (Briot et al., 2016) recent experiments using *in vitro* assays and mouse models also showed that changes in Notch activity can impact organ homeostasis in adults (Rostama et al., 2014, 2015; Briot et al., 2015). A recent research established a molecular framework coupling angiogenesis, angiocrine signals and osteogenesis via Notch signaling (Ramasamy et al., 2014).

SMC have high plasticity and are able to convert from the differentiated contractile phenotype to a variety of synthetic dedifferentiated states exhibiting in some cases chondrogenesis and osteogenesis during the pathogenesis of vascular diseases (Hilaire et al., 2016). The mechanisms of bone and vascular calcification seem to be similar and are connected through Notch/BMP/TGF-β crosstalk (Hilaire et al., 2016; Towler, 2017). It is known also that aortic tissues of BAV-patients are predisposed to progressive calcification and thus proosteogenic mechanisms might be involved in the pathogenesis of BAV-associated aortopathies.

Thus, it is obvious that functionality of SMC associated with their differentiation state is attenuated in the cells deriving from the TAA patients of both BAV and TAV groups. Myocardin, TGF-β, Notch and BMP are the main pathways responsible for the functional state of SMC in the aortic wall. The objective of the present study was to elucidate more precisely the mechanisms that attenuate differentiation state of the diseased SMC and to reveal possible differences between BAV- and TAV-derived SMC. For this we stimulated TGF-β, osteogenic and Notch differentiation pathways and compared control and diseased cells from the BAV and TAV group. We show that myocardin, TGF-β, BMP and Notch–related mechanisms of SMC differentiation are attenuated in the smooth muscle cells of the patients with thoracic aortic aneurys; Notch-dependent and proosteogenic genes show distinct expression in smooth muscle cells of BAV- vs. TAV-related aortic aneurysms.

## Materials and methods

### Patients

The clinical research protocol was approved by the local Ethics Committee of the Almazov Federal Medical Research Center and was in accordance with the principle of the Declaration of Helsinki. All patients gave signed informed consent.

Samples of the aneurysmal wall of the thoracic aorta were harvested during aortic surgery at the Almazov Federal Medical Research Center. Eleven specimens were sampled from patients with thoracic aortic aneurysm with tricuspid aortic valve (TAV) (*n* = 11) (Table 1). Fourteen specimens were sampled from patients with thoracic aortic aneurysm with bicuspid aortic valve (BAV) (*n* = 14). Patients with connective tissue disorders were excluded. Control aortic specimens were obtained from organ transplant donors (*n* = 13) and all had TAV. All tissues were sampled from the outer curvature of the thoracic aorta.

**Table 1:** Clinical characteristics of the study groups.

	**TAV (*n* = 11)**	**BAV (*n* = 14)**	**C (*n* = 13)**
Male gender (%)	46	59	60
Age (years)	64 ± 6	62 ± 4	40 ± 5
Aortic diameter (cm)	5.6 ± 0.18	5.9 ± 0.16	<3
Aortic CSA/h (cm^2^/m)	6.6 ± 0.6	7.6 ± 0.6	
Peak valve gradient (mmHg)	83 ± 9	86 ± 11	
Mean valve gradient (mmHg)	55 ± 7	59 ± 9	
Aortic valve area index (cm^2^/m^2^)	0.39 ± 0.02	0.38 ± 0.02	
Hypertension (%)	84	81	
**Medication**			
Angiotensin receptor blockers (%)	38	18	
Statins (%)	0	41	
Aspirin (%)	31	14	

*Values are means ± S.E.M*.

### Primary cultures

To obtain SMC cultures the cells were isolated from the aortic wall by collagenase digestion as described previously (Malashicheva et al., 2016). SMC were cultured in growth medium containing DMEM (Invitrogen) supplemented with 20% fetal bovine serum (FBS, Invitrogen), 2 mM L-glutamine, sodium pyruvate and penicillin/streptomycin (100 mg/l) (Invitrogen). The cells were used in experiments at passages 2–7.

### Osteogenic differentiation

To induce osteogenic differentiation SMC were seeded at 80% confluency in growth medium and after 24 h the medium was changed to osteogenic differentiation media containing DMEM/F12 (Invitrogen) with 1% penicillin/streptomycin, 10 mM β-glycerolphosphate, 200 μM L-ascorbic acid and 100 nM dexamethasone (Sigma). Gene expression was analyzed 5 days after the induction of differentiation.

### TGF-β induced differentiation

SMC were seeded in the growth medium and after they were attached to the culture plate the medium was changed to serum-free DMEM/F12 (Invitrogen) with 1% penicillin/streptomycin. After 24 h 2,5 ng/ml of human recombinant TGF-β1 (Peprotech) was added. The cells were harvested 96 h after the addition of TGF-β1 for either qPCR or Westernblotting analyses.

### Genetic construct and lentiviruses

Lentiviral packaging plasmids were a generous gift of D. Trono (École Polytechnique Fédérale de Lausanne, Switzerland); pLVTHM was modified to bear Notch1 intracellular domain of Notch1 (Kostina et al., 2016). Lentiviral particles were produced using 293 packaging line as described previously (Malashicheva et al., 2007).

The efficiency of transgene expression with NICD-bearing virus was verified by Westernblotting with the antibodies to Notch1 (SC6014, Santa Cruz) and by qPCR with primers to Notch1 target gene *HEY1*.

Control transductions were performed using GFP-bearing lentiviral vector.

### Alkaline-phosphatase staining

*In vitro* calcification was determined by alkaline-phosphatase (ALP) activity, which is an early marker of osteogenic differentiation. ALP staining was performed using Sigma BCIP®/NBT kit 14 days after the initiation of differentiation. Cells were washed with PBS and incubated with alkaline-phosphatase working solution for 10-15 min at room temperature. ALP activity appeared as blue deposition and plates were photographed with digital camera.

### qPCR analysis

Total RNA was extracted from SMCs using Extract RNA reagent (Eurogen, Russia) according to the instructions of the manufacturer. Total RNA (1 μg) was reverse transcribed with MMLV RT kit (Eurogen, Russia). Real-time PCR was performed with 1 μL cDNA and SYBRGreen PCR Mastermix (Eurogen, Russia) in the Light Cycler system using specific forward and reverse primers for target genes. Primer sequences are available upon request. Changes in target genes expression levels were calculated using the comparative ΔΔCT method. The mRNA levels were normalized to *GAPDH* mRNA.

### Westernblotting

Proteins were extracted from SMC as follows. The cells were treated with lysis buffer containing 50 mM Tris (pH 8), 150 mM NaCl, 1% Triton X-100, 1% sodium deoxycholate, and 5 mM EDTA (Sigma) and protease inhibitor cocktail (Roche). Extracts were separated by 10% sodium dodecyl sulfate-polyacrylamide gel electrophoresis (SDS-PAGE). Primary antibodies used for Westernblotting were the following: Notch1 (SC6014, Santa Cruz), SM22α (ab14106, Abcam), SMA (sc-32251, Santa Cruz), vimentin (M072529, DAKO), beta-actin (ab6276, Abcam), pSMAD (Abcam), calponin (SantaCruz), tubulin (Sigma).

### Statistics

Discriminant analysis was used to investigate what parameters in gene expression could divide groups of patients. Also discriminant analysis gives an opportunity to predict what parameters are mostly common for each group.

Linear discriminant function analysis was performed to determine which continuous variables discriminate between groups of BAV, TAV and controls. Continuous variables were qPCR gene expression data from ΔΔCT estimation. A stepwise analysis enumerating steps, *p*-value significance level, and *F*-test were performed. A discrimination level was evaluated by assessing Wilks' lambda. Significance of an identifying criterion was determined after drawing scatterplots of canonical values and calculating classification value and Mahalanobis squared distance. Discriminant function analysis was performed with Statistica 7.0 software.

Correlation analysis was performed using R software (version 2.12.0; R Foundation for Statistical Computing, Vienna, Austria). Spearman correlation coefficient was used. The significance of correlations was evaluated by a two-tailed Mann–Whitney test.

qPCR data on gene expression was analyzed using Graph Pad Prizm. Values are expressed as means ± SEM. Groups were compared using the Mann–Whitney non-parametric test. A value of *P* ≤ 0.05 was considered significant.

## Results

### BAV- and TAV-derived SMC demonstrate distinct correlation profiles for expression of myocardin

**Figure 1:**
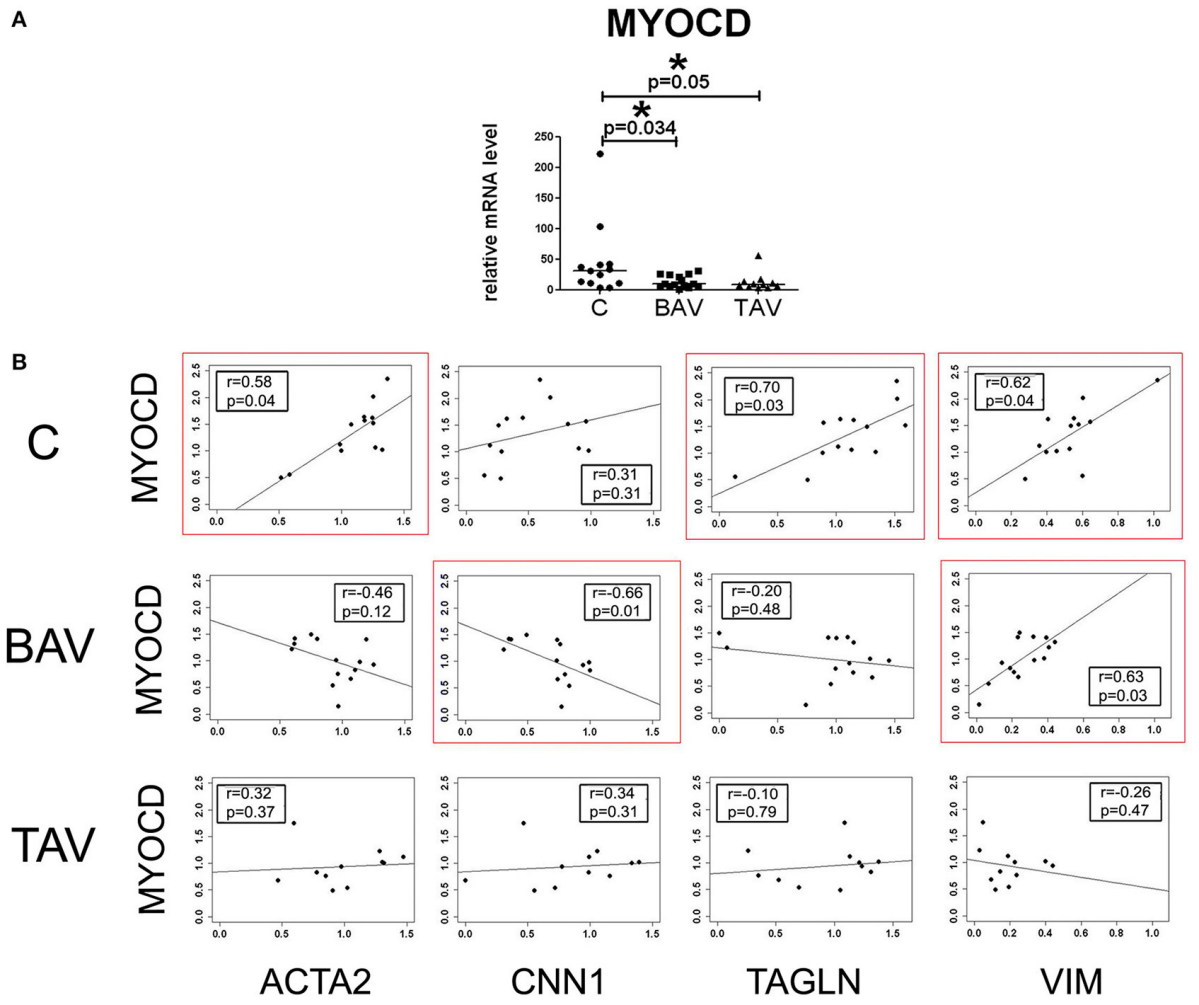
Myocardin expression in aortic smooth muscle cells (SMC) from patients with thoracic aortic aneurysm with tricuspid aortic valve (TAV) or bicuspid aortic valve (BAV) and controls (C). **(A)** Relative mRNA levels for *MYOCD* (myocardin) in SMC from patients with aortic aneurysm (TAV, *n* = 11; BAV, *n* = 14), and control cells (C, *n* = 13). The significance of gene expression differences between groups was evaluated by a two-tailed Mann–Whitney test; line represents the median; ^*^p < 0.05. **(B)** Scatter plots showing correlations of *MYOCD* and SMC marker genes *ACTA2, TAGLN, CNN1, VIM* expression levels in SMC of patients with aortic aneurysm with BAV, TAV, and healthy controls. mRNA levels were analyzed by qPCR and normalized to *GAPDH*. The x- and y-axis show log RQ (relative quantification = 2^−ΔΔCt^). *r*, Spearman correlation coefficient; p, *p*-value. *P*-values less than 0.05 were considered to be statistically significant. Statistically significant correlations are highlighted by red rectangles.

We measured the expression level of *MYOCD* gene encoding myocardin in smooth muscle cells (SMC) derived from aortic wall of the patients with thoracic aortic aneurysm (TAA) associated with either tricuspid- or bicuspid aortic valve (TAV- and BAV- correspondingly) and healthy controls. *MYOCD* expression level was significantly down regulated in the SMC of both groups of the patents (Figure 1A). To explore the relationships between myocardin and smooth muscle-specific contractile proteins we assessed correlations between mRNA transcript levels of *MYOCD* and SMC differentiation marker genes *ACTA2* (SMA), *TAGLN* (SM22), *CNN1* (calponin), and *VIM* (vimentin) using the Spearman correlation coefficient (Figure 1B). MYOCD expression level positively and statistically significantly correlated with *ACTA2, TAGLN*, and *VIM* expression levels in control group. In contrary, in the BAV group we observed statistically significant positive correlation only between *MYOCD* and *VIM* expression levels and negative correlation between *MYOCD* and *CNN1*. In the TAV group no correlations were observed. Thus, the patterns of correlations of *MYOCD* expression with contractile proteins expression were distinct in all three groups of SMC: BAV- and TAV-derived ad controls.

### Expression analysis of contractile, BMP- and notch and related gene in SMC of BAV and TAV patients

**Figure 2:**
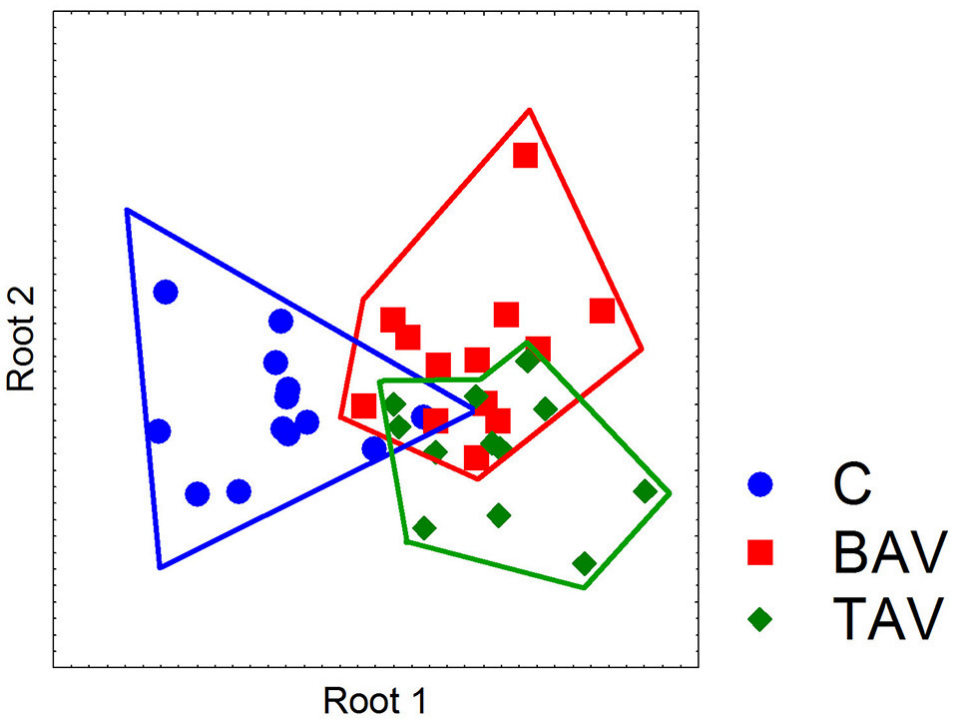
Partition of the examined groups based on the results of discriminant analysis. Discriminant analysis was used to investigate what parameters in gene expression could divide groups of patients. Expression data on SMC differentiation markers (*MYOCD, ACTA2, CNN, TAGLN*, and *VIM*), Notch-related genes (*NOTCH1, NOTCH2, NOTCH3, JAG1, HES1, HEY, SNAIL*, and *SLUG*) and proosteogenic genes (*BMP2, RUNX2, POSTIN, CTNNB1, SOX9, OPN*, and *OPG*) were used. In the course of discriminant analysis 11 genes with high prognostic and discriminant value were identified (Table 2) and these genes were used to examine if the SMC from BAV, TAV, and control groups form independent clusters. Dot plot demonstrates that control SMC form a separate cluster from both BAV- and TAV groups by expression of Notch-related and proosteogenic genes, while BAV- and TAV-derived SMC clusters partially overlap.

To identify if the SMC from BAV- and TAV-patients and control group form clusters by gene expression we used multivariate discriminant function analysis. In addition to *MYOCD, ACTA2, CNN, TAGLN, VIM* expression data from above section we measured the expression of Notch-related genes known to be expressed in SMC (*NOTCH1, NOTCH2, NOTCH3, JAG1, HES1, HEY, SNAIL*, and *SLUG*) and proosteogenic genes known to be involved in calcification (*BMP2, RUNX2, POSTIN, CTNNB1*, SOX9, *OPN*, and *OPG*; Rutkovskiy et al., 2016). In the course of discriminant analysis we identified 11 genes with high prognostic and discriminant value (Table 2). These genes were used to examine if the SMC from BAV, TAV, and control groups form independent clusters. Graphical result of the examined groups based on the discriminant analysis (Figure 2) demonstrates that control SMC form a separate cluster from both BAV- and TAV groups by expression of Notch-related and proosteogenic genes, while BAV- and TAV-derived SMC clusters demonstrate distinct patterns with partial overlap.

**Table 2:** Genes included in the model for discriminant analysis.

	**Wilks' Lambda**	**Partial Lambda**	**F-remove (2.71)**	***p*-level**
VIM	0.293439	0.787751	3.367953	0.050683
BMP2	0.258843	0.893039	1.497141	0.243155
SNAIL	0.308272	0.749846	4.170081	0.027362
NOTCH1	0.231626	0.997973	0.025387	0.974958
HEY1	0.238413	0.969564	0.392391	0.679527
TAGLN	0.258818	0.893125	1.495798	0.243447
ACTA2	0.374827	0.616702	7.769094	0.002377
CNN1	0.376774	0.613516	7.874362	0.002228
MYOCD	0.284798	0.811652	2.900690	0.073642
SLUG	0.271318	0.851977	2.171758	0.135005
OPG	0.270577	0.854312	2.131650	0.139704

### TGF-beta induces similar response in BAV and TAV-derived SMC

**Figure 3:**
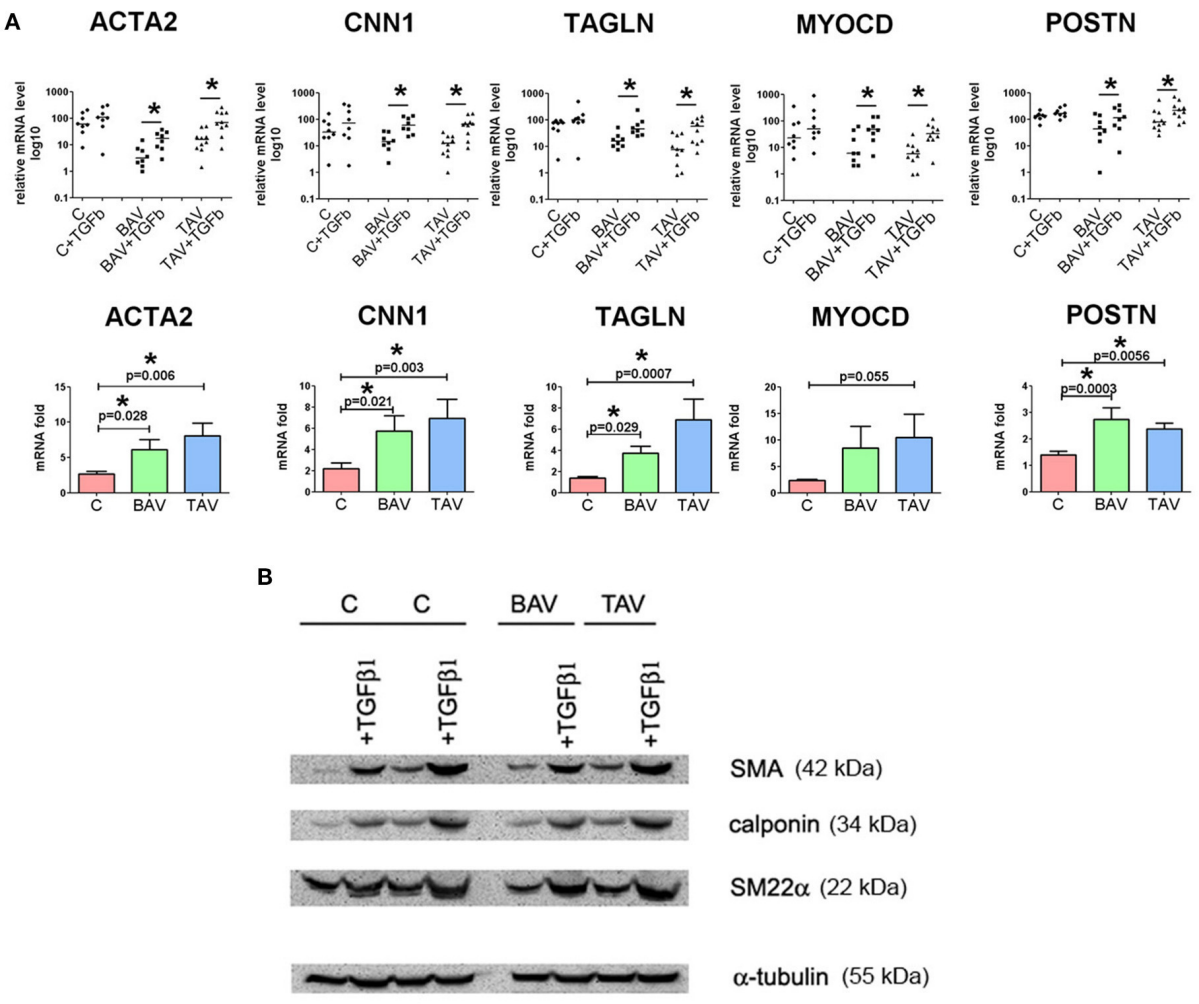
Effects of transforming growth factor TGFβ1 stimulation on the expression of differentiation marker genes in primary human aortic smooth muscle cells (SMC) from healthy donors (C) and patients with thoracic aortic aneurysm with either tricuspid aortic valve (TAV) or bicuspid aortic valve (BAV). SMC were grown to postconfluence, serum-starved for 96 h, and then TGFβ1 (2,5 ng/ml) was added for 96 h. **(A)** Upper panel: as confirmed statistically, TGF-β1 treatment significantly increased mRNA expression for SMC differentiation marker genes *ACTA2* (α-smooth muscle actin), *TAGLN* (transgelin, or SM22α), *CNN1* (calponin), *MYOCD* and *POSTN* in SMC from the patients with thoracic aortic aneurysm. Line represents the median; ^*^p < 0.05. Lower panel: fold change gene expression for *ACTA2, TAGLN, CNN1, MYOCD*, and *POSTN* in SMC from controls and patients with thoracic aortic aneurysm after treatment with TGF-β1. Data are presented as fold change mRNA i.e., the ratio of mRNA level after TGF-β1 treatment to the initial mRNA level before the treatment. Bar represents the mean with SD. mRNA level was determined by qPCR and gene expression was equalized by *GAPDH* expression. C, *n* = 8; TAV, *n* = 10; BAV, *n* = 8. Groups were compared using Mann-Whitney nonparametric test. **(B)** Westernblotting analysis for detection of smooth muscle actin (SMA), calponin, SM22α in SMC after treatment with TGF-β1. α-Tubulin was used as a control to verify total amount of protein.

In order to define whether there was a difference in TGF-β responsiveness between BAV- and TAV-derived SMC and controls, we measured the expression of phenotypic markers of the contractile SMC after treatment with human recombinant TGF-β1 (Figure 3). *ACTA2, CNN1, TAGLN, MYOCD, POSTN* mRNA were up regulated after TGF-β treatment in all three groups (Figure 3A, upper panel). We correspondingly observed elevation of protein level of SMA, calponin, SM22α in all three groups of SMC as well (Figure 3B). However, when compared to SMC from healthy aortas, aneurysm-derived SMC exhibited a more pronounced TGF-β induced response, which could be counted more precisely by qPCR data. In the lower panel of Figure 3A, we presented data as fold change mRNA i.e., the ratio of mRNA level after TGF-β treatment to the initial mRNA level before the treatment. The transcriptional upregulation was more pronounced in BAV- and TAV-derived SMC, respectively, in comparison with control: *ACTA2* (6.1- and 8.1-fold, vs. 2.7-fold), *TAGLN* (3.7- and 7.2-fold, vs. 1.4-fold), *CNN1* (5.7- and 7-fold, vs. 2.2-fold), *POSTN* (2.7- and 2.4-fold vs. 1.4-fold). Although the data on *MYOCD* mRNA expression did not achieve statistical significance, there was a trend toward more powerful induction of its mRNA in SMC from TAV patients comparing to the control group (*p* = 0.055). Thus, TGF-beta induces similar pattern of responsive gene activation for BAV and TAV-derived cells, and this activation is higher in the cells of BAV and TAV patients compared to the cells of healthy donors.

### BAV- and TAV-derived smooth muscle cells differ in their differentiation properties in osteogenic conditions

**Figure 4:**
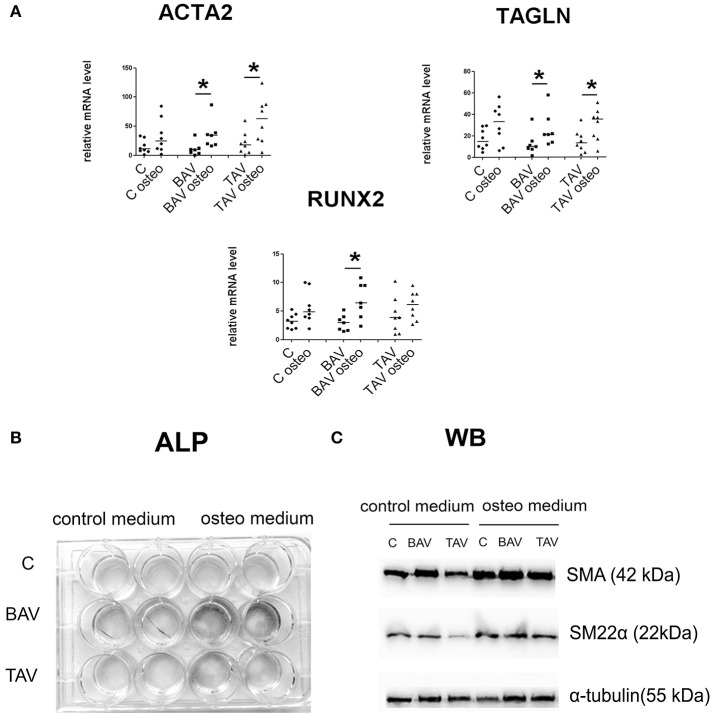
BAV- and TAV-derived smooth muscle cells (SMC) differ in their differentiation properties in osteogenic conditions. SMC from patients with thoracic aortic aneurysm with either tricuspid aortic valve (TAV) or bicuspid aortic valve (BAV) and controls (C) were cultured with control or osteogenic [in the presence of 10 mM β-glycerolphosphate, 200 μM L-ascorbic acid and 100 nM dexamethasone (Sigma)] medium for 5 days. At day 5, total RNA and protein were extracted for qPCR analysis **(A)** and Westernblotting **(B)**, respectively. TAV, *n* = 8, BAV, *n* = 7, C, *n* = 8. **(A)** Culture in osteogenic conditions was accompanied by SMC-marker gene activation, which is stronger in aneurysm-derived cells, and also by statistically significant increase in *RUNX2* expression only in BAV-derived cells. Relative mRNA levels for *ACTA2, TAGLN*, and *RUNX2* in human aortic SMC were determined by qPCR and normalized to *GAPDH*. Groups were compared using Mann-Whitney nonparametric test; line represents the median; ^*^p < 0.05. **(B)** *In vitro* induced calcification. SMC were cultured in osteogenic conditions, and ALP activity was visualized with BCIP/NBT at day 14. **(C)** Representative Western blots for detection of smooth muscle actin (SMA), and SM22α, in human aortic SMC after culture in osteogenic conditions. α-Tubulin was used as a control to verify total amount of protein.

We induced osteogenic differentiation in SMC by addition of specific osteogenic factors to culture medium (Figure 4). Protein levels for SMA and SM22 were elevated in all three groups, by Westernblotting staining (Figure 4C). More precise estimation of corresponding gene transcription levels revealed statistically significant elevation of *ACTA2* and *TAGLN* transcript levels in both groups of diseased cells, but not in control group (Figure 4A). Osteogenic medium induced significant induction of *RUNX2* transcription in BAV-derived SMC after 5 days of osteogenic induction (Figure 4A). To verify if osteogenic medium indeed caused change in SMC phenotype, we cultured the cells under osteogenic conditions for 14 days and then stained them for alkaline phosphatase activity (ALP), a marker of osteogenic differentiation. Correspondingly we observed the most intensive alkaline phosphatase activity (ALP), for the SMC from BAV-patients (Figure 4B) suggesting that BAV-derived aortic SMC are more prone to osteogenic differentiation compared to TAV-derived SMC and SMC from healthy controls.

### Notch differently induces differentiation in BAV-derived SMC

**Figure 5:**
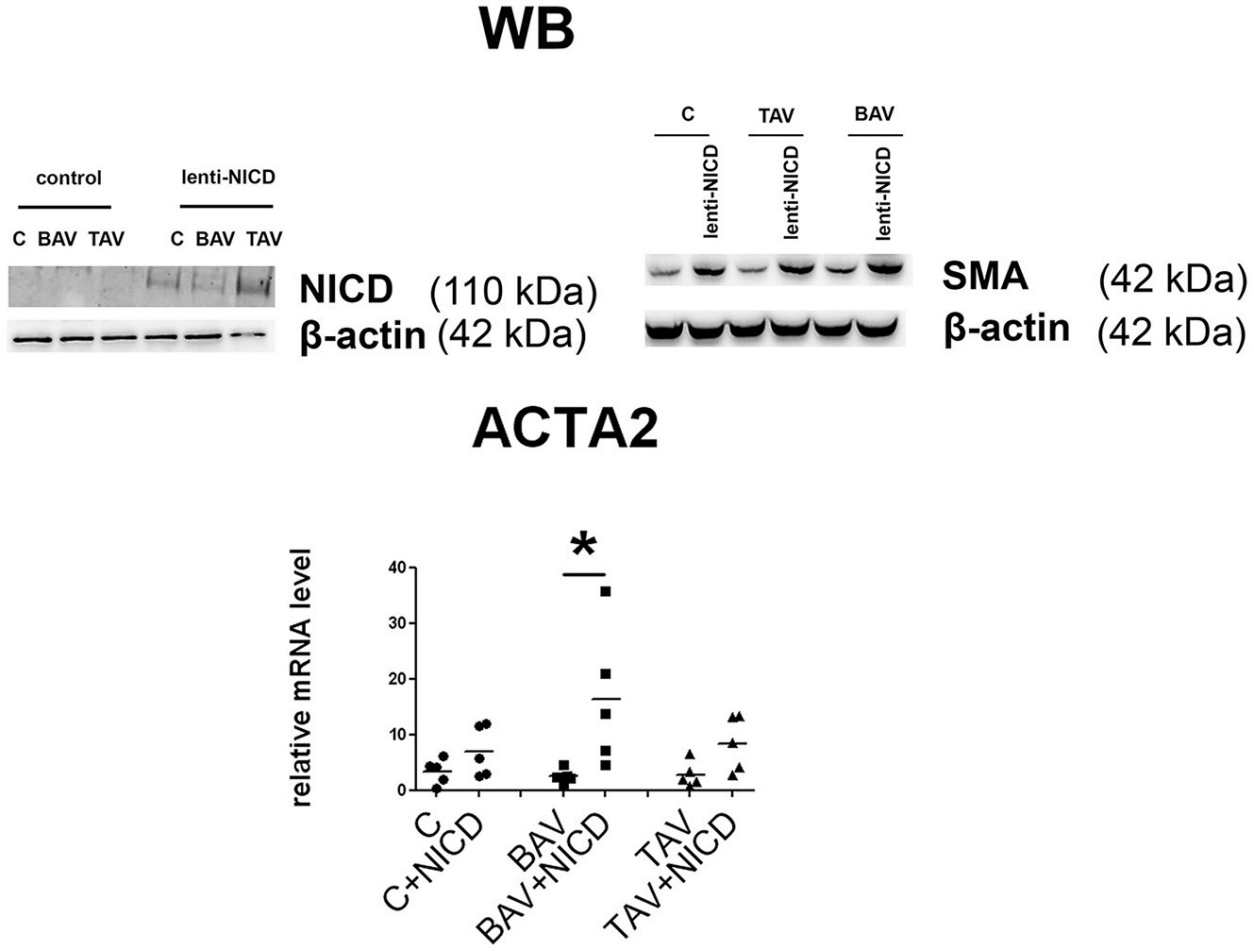
Induction of smooth muscle differentiation in primary aortic smooth muscle cells (SMC) from healthy donors (C) and patients with thoracic aortic aneurysm either tricuspid aortic valve (TAV) or bicuspid aortic valve (BAV) by introduction of Notch-intracellular domain (NICD). C, *n* = 5; BAV, *n* = 5; TAV, *n* = 5. Upper panel represents Western blots for detection of Notch1-activated intracellular domain (NICD) and SMA accumulation after lenrivirial transduction with NICD. Lower panel represents analysis of *ACTA2* induction. mRNA level was determined by qPCR and gene expression was equalized by *GAPDH* expression. Groups are compared using Mann-Whitney nonparametric test; line represents the median; ^*^p < 0.05.

To look how responsive to Notch activation are the SMC derived from BAV- and TAV-patients compared to control cells we activated Notch signaling in SMC via transduction with the lentivirus bearing Notch1-intracellular domain (NICD) (Figure 5). For control GFP-bearing virus was used. Notch-activation induced *ACTA2* transcription and corresponding SMA protein accumulation, which was observed in all three groups, but statistically significant *ACTA2* up regulation was observed only in BAV group. The data suggests that BAV-derived cells are more responsive to Notch activation of *ACTA2* transcription comparing to TAV and control cells.

### Notch signaling is coupling osteogenic and myofibroblast induction in SMC

**Figure 6:**
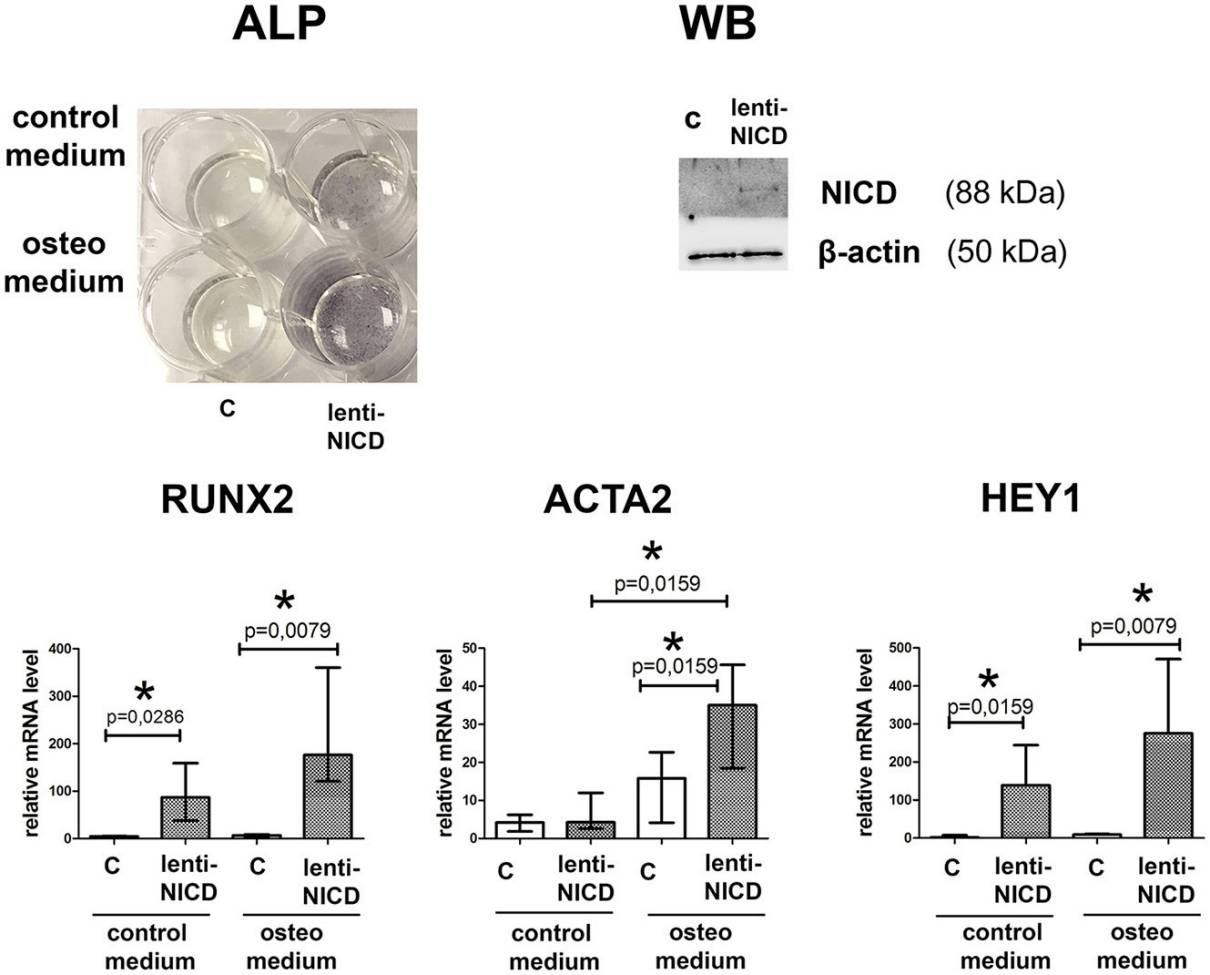
Notch activation in smooth muscle cells (SMC) causes proosteogenic response. SMC were cultured in the presence of growth medium or osteogenic medium and with absence or presence of NICD (c and lenti-NICD, respectively). **Upper panel** represents ALP activity revealed in SMC 14 days after the induction of osteogenic differentiation. Western blot (WB) represents detection of Notch1-activated intracellular domain (NICD) in the transduced SMC. **Lower panel** represent analysis of *RUNX2, ACTA2*, and *HEY1* induction by NICD. Level of mRNA was determined by qPCR and gene expression was equalized by *GAPDH* expression. Groups are compared using Mann-Whitney nonparametric test; bars represent SEM, ^*^p < 0.05.

Participation of Notch signaling in osteogenic differentiation is rather controversial as this signaling has been shown both to promote and to prevent proosteogenic gene expression (Acharya et al., 2011; Yip et al., 2011; Zeng et al., 2013; Theodoris et al., 2015). Therefore, we induced osteogenic differentiation in aortic SMC and activated Notch signaling via lentivitral transduction with NICD-bearing virus (Figure 6). ALP staining after 14 days of culture in osteogenic conditions showed that NICD increased calcification of SMC in the presence of osteogenic medium comparing to the osteogenic medium without NICD. NICD induced up regulation of proosteogenic *RUNX2* transcription and Notch target gene transcription *HEY1* in both normal and osteogenic medium. In contrary *ACTA2* transcription was significantly induced by NICD only in osteogenic conditions (Figure 6).

## Discussion

In this study we show that smooth muscle cell differentiation pathways that are dependent on myocardin and TGF-β are attenuated in the patients with thoracic aortic aneurysm associated with bicuspid or tricuspid aortic valve. BAV-derived SMC differ from TAV-derived SMC in sensitivity to Notch-activation and to proosteogenic signals.

Consistent with the previous study that smooth muscle cells from thoracic aneurysms are characterized by decreased expression of SMC contractile proteins (Malashicheva et al., 2016), both BAV- and TAV-derived smooth muscle cells also demonstrated reduced *MYOCD expression level* compared to the cells from healthy individuals. Moreover, in SMC from healthy aortas *MYOCD* level positively correlated with the levels of *ACTA2* and *TAGLN*, which are early SMC markers, but not with later marker *CNN1*, which is activated in the middle stage of the SMC differentiation (Owens et al., 2004). At the same time, BAV-derived SMC demonstrated a strong negative correlation between *MYOCD* and *CNN1* expression, while in TAV-derived SMC we did not observe any correlation between MYOCD and other transcripts.

Myocardin is important in maintaining the normal SMC contractile phenotype (Long et al., 2008; Miano, 2015). The exact relation of myocardin upregulation to the heart and vascular diseases remains unclear. Remarkably, mutations in the *MYH11* (smooth muscle myosin heavy chain) and *ACTA2* (smooth muscle actin) genes (Zhu et al., 2006; Guo et al., 2007) were shown to cause familial thoracic aneurysms and dissections. Each of these genes is regulated directly by myocardin in the postnatal vasculature confirming that dysregulated myocardin-dependent transcription can be directly involved in aneurysm formation in humans (Huang et al., 2008).

To our surprise, we revealed a direct correlation of myocardin mRNA with vimentin mRNA (VIM) in control and BAV-derived SMC (Figure 1), but not in TAV-derived cells. Vimentin is a cytoskeletal protein of the intermediate filaments. Increased expression of vimentin in the vascular SMC has been associated with vascular injury and used as a marker of SMC synthetic, dedifferentiated phenotype (Owens et al., 2004). However, it is apparent, that functions of vimentin are more complex, and vimentin is a necessary component of fully functional contractile apparatus in smooth muscle cells.

Our data suggest that the observed “imbalance” between myocardin and smooth muscle-specific genes expression in aortic SMC from BAV and TAV-associated aneurysms is likely to depend on additional still unknown signals that can modulate a role of myocardin in directing an endogenous SMC differentiation program in aneurysm and this requires further research.

TGF-β is among the most potent factors that promote and maintain the vascular SMC contractile phenotype by upregulating smooth muscle structural genes. We revealed that aortic SMC from BAV- and TAV-related aneurysms are more responsive to pro-differentiation effect of the exogenous TGF-β1 than SMC from healthy aortas. However, we did not observe any difference in the response to TGF-β1 between BAV- and TAV-derived SMC. TGF-β pathway is apparently involved in TAA progression. Accumulating evidences are associated with increased TGF-β signaling in aneurysms (Forte et al., 2013; Gomez et al., 2013; Nataatmadja et al., 2013). Recently increased TGF-β activity during TAA formation has been reported in patients with TAV, but not BAV, presumably as a result of an increased sequestration of TGF-β in the extracellular matrix in aortas of BAV patients (Paloschi et al., 2015). Evidently, TGF-β signaling in aneurysm progression is very complex and is also influenced by epigenetic mechanisms such as histone modifications, microRNA expression and possibly others (Forte et al., 2016; Albinsson et al., 2017).

The discriminant analysis of expression of genes responsible for SMC differentiation state and genes related to Notch and proosteogenic regulation (Figure 2) suggested that SMC from BAV and TAV patients have distinct gene expression profile of these groups of genes from the cells of healthy donors.

Notch is an important regulator of SMC (Boucher et al., 2012). Among all Notch receptors and ligands Notch2 and Notch3 appear to be the most important for SMC. These two receptors influence the phenotype and functions of SMC (Baeten and Lilly, 2015; Liu et al., 2015). Notch is a key signaling pathway in development, ensuring cross talk between different types of cells and their physiological differentiation (Andersson et al., 2011). Notch signaling in the endothelium of the vessel is positioned to mediate differentiation of underlying SMC ensuring integrity of the vessel wall (Pedrosa et al., 2015). We have shown recently that Notch signaling is attenuated in aortic endothelial cells of BAV patients (Kostina et al., 2016). In the present work we show alteration in Notch responsiveness in BAV-derived SMC. We suggest that the initial process of the vessel formation as well as further healing in response to constant shear stress in the aorta could be impaired in the BAV patients via Notch-dependent events in particular through inactive feedback loop connecting endothelial and smooth muscle cells and their mutual influence on cellular functional state.

Cardiovascular calcification is gaining an increased attention nowadays as it seems to be a “default” state of various pathologies (Hilaire et al., 2016). Osteogenic medium induced more elevated *RUNX2* expression and more prominent calcification in BAV-derived SMC. These results suggest a dysregulation of proosteogenic pathways in the BAV-derived cells. Notch activation induced significant elevation of *ACTA2* expression and corresponding SMA protein accumulation in BAV-derived cells. It has been shown previously that the cross-talk between BMP2 and Notch signaling pathways induces osteogenic differentiation of vascular SMC (Shimizu et al., 2011). At the same time increased expression of *ACTA2* has been shown to directly reduce the clonal potential of human mesenchymal stem cells and to guide their differentiation toward osteoblasts. Hence, SMA not only identifies osteoprogenitors in mesenchymal populations as shown by others before (Kalajzic et al., 2008; Grcevic et al., 2012), but may be part of the mechanism driving differentiation (Talele et al., 2015). Our data are in line with this as we observed that only BAV-derived cells are able to elevate the level of *ACTA2* in response to Notch stimulation. In addition we show that simultaneous action of osteogenic medium and Notch activation leads to elevated *ACTA2* expression and to an increase in osteogenic differentiation in SMC. Thus, this Notch-dependent elevation of *ACTA2* might be a direct prerequisite to calcification. Synergy between attenuated Notch and proosteogenic sensitivity could cause the final predisposition to osteogenic phenotype in BAV-derived SMC.

This study has some important limitations. First, the groups of the patients were not quite large. Second, we worked *in vitro* with isolated smooth muscle cells, but in aortic wall there is a community of SMC and endothelial cells and the communication between these different type of cells now is gaining more attention to understand proper function of the whole vessel (Lilly, 2014). Nevertheless, we suggest that our findings are relevant for searching potential targets to ameliorate aortic wall integrity.

## Author contributions

EI and DK made experiments, analyzed data and wrote the manuscript. OI, VU, NG, and OM analyzed data of the patients and collected patient samples. AG and AK analyzed data. AM designed research, analyzed data, and wrote the manuscript.

### Conflict of interest statement

The authors declare that the research was conducted in the absence of any commercial or financial relationships that could be construed as a potential conflict of interest.
